# Endovascular Creation of Native Arteriovenous Fistulas for Hemodialysis: A Percutaneous, Vessel-Sparing Strategy for Vascular Access

**DOI:** 10.3390/jcm15051855

**Published:** 2026-02-28

**Authors:** Giulio Distefano, Alessio Sturiale, Concetto Sessa, Ivana Maria Grazia Alessandrello, Andrea Boncoraglio, Elisa Cicero, Dario Galeano, Roberta Maria Messina, Vincenzo Ficara, Fiorenza Rauseo, Alessia Tigano, Viviana Scollo, Fortunata Zirino, Carmelo Zuppardo, Domenico Patanè, Walter Morale

**Affiliations:** 1 Institute of Nephrology and Dialysis, Maggiore Hospital of Modica, ASP Ragusa, 97100 Ragusa, Italy; alessio.sturiale@asp.rg.it (A.S.); concetto.sessa@asp.rg.it (C.S.); ivana.alessandrello@asp.rg.it (I.M.G.A.); elisa.cicero@asp.rg.it (E.C.); dario.galeano@asp.rg.it (D.G.); robertamaria.messina@asp.rg.it (R.M.M.); vincenzo.ficara@asp.rg.it (V.F.); fiorenza.rauseo@gmail.com (F.R.); alessia.tigano@asp.rg.it (A.T.); viviana.scollo@asp.rg.it (V.S.); fortunata.zirino@asp.rg.it (F.Z.); carmelo.zuppardo@asp.rg.it (C.Z.); walter.morale@asp.rg.it (W.M.); 2 Radiology Unit, Giovanni Paolo II Hospital, ASP Ragusa, 97100 Ragusa, Italy; andrea.boncoraglio@asp.rg.it; 3 Department of Radiology, Azienda Ospedaliera Cannizzaro, 95021 Catania, Italy; domenicopatane.dp@gmail.com

**Keywords:** endovascular arteriovenous fistula, percutaneous AVF creation, hemodialysis, vascular access, native arteriovenous fistula, Ellipsys, WavelinQ

## Abstract

Surgically created native arteriovenous fistulas (AVFs) remain the preferred vascular access for chronic hemodialysis, yet they are limited by substantial early failure and progressive consumption of venous capital. Endovascular arteriovenous fistulas (endoAVFs, also referred to as percutaneous AVFs) have become a catheter-based alternative to surgical AVF (sAVF). We conduct an updated narrative, practice-oriented review of the literature on endoAVF creation, and we qualitatively synthesize evidence. Two devices are currently available in contemporary clinical practice: a dual 4 Fr-catheter, fluoroscopy-guided radiofrequency system (WavelinQ) and a single 6 Fr-catheter, ultrasound-guided thermal resistance system (Ellipsys). Across prospective studies and real-world series, endoAVF creation is consistently reported to have high technical success, with low major complication and infection rates. Clinical usability can often be achieved within weeks when ultrasound-based surveillance and protocol-driven maturation assistance are implemented; however, adjunctive procedures are frequently required and should be anticipated in program planning and informed consent. Observational comparisons and pooled analyses indicate broadly comparable functional outcomes versus surgery in selected cohorts, while estimates of primary patency and maintenance burden vary substantially across studies. Overall, endoAVFs represent a feasible, minimally invasive, vessel-sparing option that can be integrated into multidisciplinary access pathways in anatomically suitable candidates and experienced centers, complementing rather than replacing surgical strategies within a distal-first plan. Recent society practice guidance further emphasizes standardized mapping, expectation setting, troubleshooting algorithms for non-maturation, and dialysis-unit cannulation training to support consistent implementation. Pragmatic comparative studies and long-term registries using standardized endpoints, paired with healthcare-system-specific economic analyses, are needed to better define durability, resource use, and patient-centered outcomes over the full-access life cycle.

## 1. Introduction and Aim

Native arteriovenous fistulas (AVFs) are the gold standard for vascular access in chronic hemodialysis because they provide superior long-term patency and lower infection rates compared with prosthetic grafts and central venous catheters [1,2]. Despite these advantages, surgical AVF creation is burdened by substantial early failure rates, estimated around 20–50%, mainly due to the lack of maturation or development of juxta-anastomotic stenosis [3,4,5]. In this context, endovascular arteriovenous fistulas (endoAVFs) created on native vessels have emerged as a percutaneous alternative designed to reproduce the hemodynamic and anatomic characteristics of autogenous fistulas [6] in patients with favorable vascular anatomy. EndoAVF systems, under ultrasound and/or fluoroscopic guidance, create communication between adjacent arteries and veins in the proximal forearm or upper arm, with limited tissue trauma and short procedure times [7,8]. Two device platforms with different technical principles are currently used in contemporary practice: a 4-Fr dual-catheter system using radiofrequency energy (WavelinQ, BD Bard) and a 6-Fr single-catheter system based on thermal resistance anastomosis (Ellipsys, Medtronic) [9,10,11]. From first in-human feasibility series through pivotal trials, registries, and comparative cohorts, a substantial body of data has accumulated over a relatively short time, suggesting that endoAVFs achieve technical success and clinical usability that are at least comparable to contemporary surgically created native AVFs [12,13,14,15]. However, the rapidly expanding literature remains heterogeneous in terms of patient selection, access anatomy, surveillance protocols, and endpoint definitions, which can make translation into day-to-day program planning challenging [16,17,18]. In particular, clinicians often need practical guidance on how endoAVFs fit within a distal-first access strategy, what maturation assistance to anticipate, and which outcomes are most robust across different study designs. Therefore, the objective of this narrative review is to synthesize current evidence on endoAVF creation and translate it into pragmatic, practice-oriented considerations on devices and techniques, maturation pathways and reinterventions, complications, organizational/economic aspects, and the positioning of endoAVFs relative to surgical AVFs within contemporary access planning.

## 2. Methods

This is an up-to-date narrative review; no formal systematic methodology or meta-analysis was applied. Literature was identified through a targeted search of PubMed covering the period from January 2002 to December 2025. Search terms were used in different combinations and included: “endovascular arteriovenous fistula”, “percutaneous arteriovenous fistula”, “Ellipsys”, “WavelinQ”, “hemodialysis access”, “native fistula”, and “dialysis vascular access”.

We prioritized clinical studies reporting outcomes of endoAVF creation (prospective or retrospective cohorts, registries, and comparative studies). We also considered systematic reviews/meta-analyses and selected practice guidance or methodological papers relevant to dialysis access reporting. Additional records were identified by screening reference lists of key articles and guidance documents.

From eligible sources, we extracted and qualitatively summarized information on device techniques and workflows, technical success and usability, patency and reinterventions (including maturation assistance), complications, and organizational/economic considerations. We did not perform a PRISMA-style study selection, formal risk-of-bias assessment, or quantitative pooling. The terms endoAVF and pAVF are often used interchangeably; we use “endoAVF” throughout while acknowledging “pAVF” as a common equivalent term.

## 3. Devices and Techniques

### 3.1. WavelinQ 4-Fr Radiofrequency System (Fluoroscopic-Guided)

**Figure 1:**
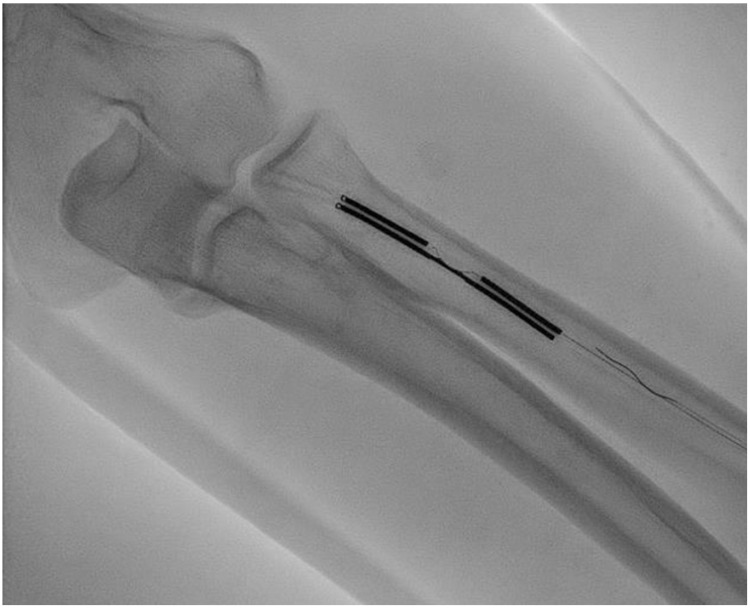
Intra-procedural angiography acquired during endoAVF creation using the WavelinQ system shows, in the proximal third of the forearm, correct alignment of the venous and arterial catheters.

**Figure 2:**
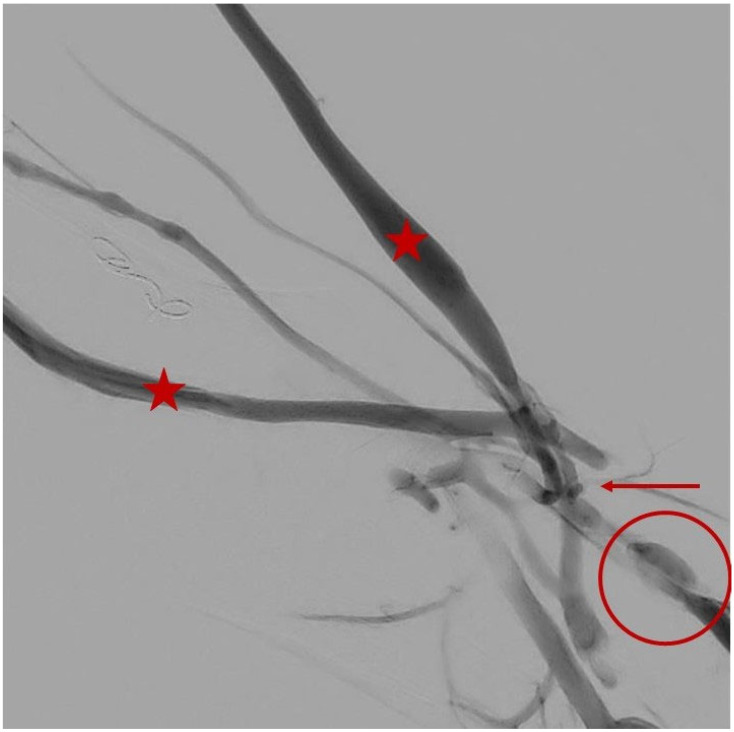
Final completion angiography performed at the end of an endoAVF creation procedure using the WavelinQ system demonstrates technical success, with optimal opacification of the anastomosis (circle), the perforating vein (arrow), and the superficial venous circulation with cephalic and basilic venous outflow in the upper arm (asterisk).

The WavelinQ system is one of the catheter-based platforms introduced for endovascular AVF creation. It uses two 4-Fr catheters with magnetic elements at their distal tips, inserted respectively into an artery (radial or ulnar) and the corresponding paired veins (venae comitantes) [8]. The procedure is typically performed under local anesthesia with ultrasound and fluoroscopic guidance. A 0.014″ guidewire is advanced into the radial or ulnar artery, over which the arterial catheter is introduced. The venous catheter is inserted into the ipsilateral comitant vein. Magnetic bands at the distal tips allow precise alignment of the two catheters at the target segment for anastomosis (Figure 1). Once alignment is confirmed, a radiofrequency pulse is delivered to create side-to-side communication between the two vascular lumens, resulting in endoluminal anastomosis (Figure 2). At the end of the procedure, coil embolization of one of the paired deep veins may be performed to divert flow toward the superficial venous system via the perforating vein, facilitating a cannulable outflow circuit [8]. In prospective clinical experience, technical success has been high, and suitability for dialysis use has been achieved in a substantial proportion of cases within first months after creation [12]. Procedure times in contemporary series are typically within a range compatible with outpatient workflows [12].

### 3.2. Ellipsys 6-Fr Thermal Resistance System (Ultrasound-Guided)

**Figure 3:**
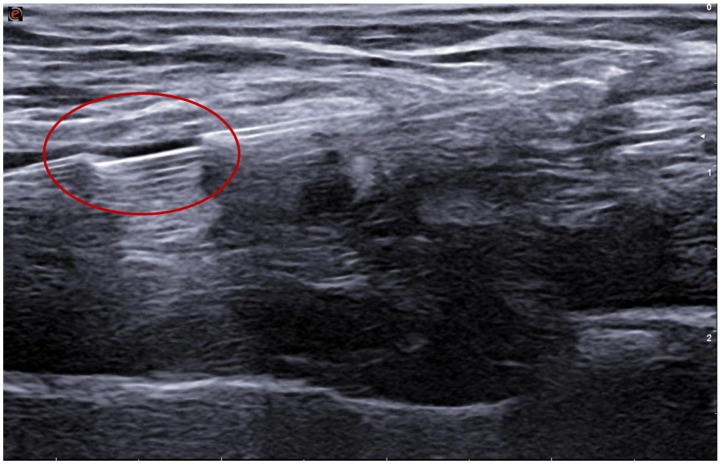
Intra-procedural ultrasound image acquired during endoAVF creation using the Ellipsys system shows the 6-Fr device as the anchoring mechanism is retracted to capture the venous and arterial walls involved in the anastomosis.

**Figure 4:**
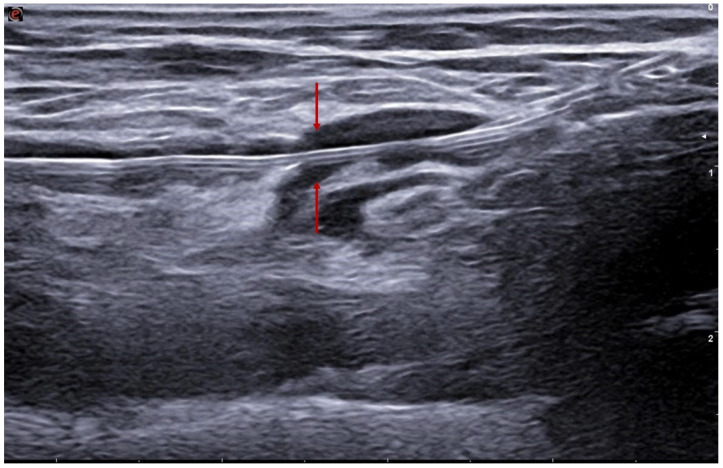
Imaging acquired immediately after energy delivery demonstrates successful creation of the anastomosis (arrows).

**Figure 5:**
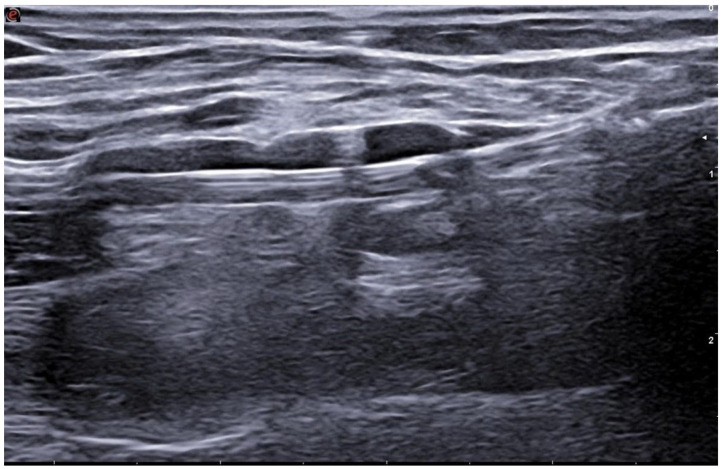
Under ultrasound guidance, the anastomosis is remodeled using 5 mm balloon angioplasty.

**Figure 6:**
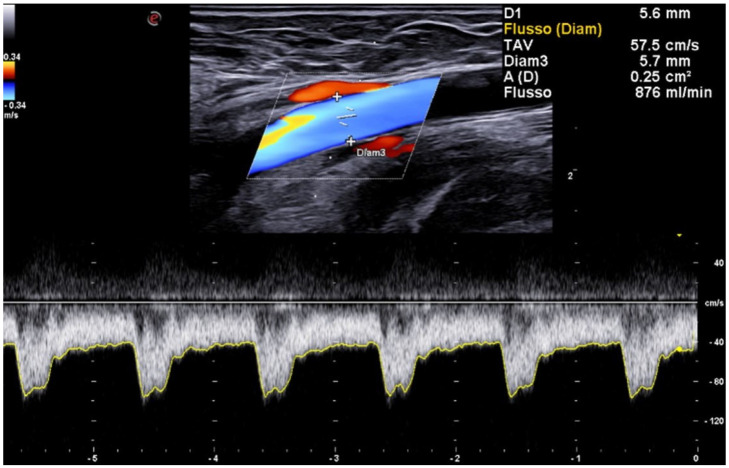
Post-procedural duplex ultrasound with spectral Doppler assessment performed at the end of endoAVF creation using a 6-Fr device confirms technical success, demonstrating a low-resistance waveform and satisfactory flow volume.

The Ellipsys system employs a single over-the-wire 6-Fr catheter introduced via venous access to the forearm perforating vein and advanced to the level of the proximal radial artery [13,14]. The procedure begins with a micropuncture set advanced under real-time ultrasound guidance along the axis of the perforating vein. The needle then traverses the wall of the proximal radial artery, allowing a guidewire to be passed from the vein into the artery. The Ellipsys catheter is advanced over the wire into the radial artery. A distal anchoring mechanism secures the arterial and venous walls and permits creation of the anastomosis by thermal resistance (Figure 3 and Figure 4). The entire procedure is performed under ultrasound guidance alone, without fluoroscopy or iodinated contrast [14]. The main advantages include the absence of ionizing radiation, elimination of contrast-induced nephrotoxicity risk, and the possibility of performing the procedure in an outpatient or nephrology-based setting. In the pivotal multicentre trial, the Ellipsys system achieved a 95% technical success rate, with a median procedure time of 28 min, and a very low incidence of peri-procedural complications [11]; long-term follow-up of the pivotal cohort reported durable access performance throughout 5 years, with high cumulative and functional patency and no major pAVF-related complications during long-term follow-up [8]. Longer-term registries report cumulative patency > 80% at 12 months, with relatively low reintervention rates [13,19]. Angioplasty of the anastomosis and the perforating vein with balloons (often up to 5 mm) is incorporated into Ellipsys-based protocols to promote remodeling of the venous circuit (Figure 5 and Figure 6) [14].

Beyond these device-specific steps, several practical considerations apply to both endoAVF platforms: both devices share a relatively short learning curve when adopted by operators already experienced in dialysis access interventions [8]. In routine practice, careful pre-procedural ultrasound mapping (arterial and venous diameter, depth, course, presence of stenoses or central vein disease) is essential for case selection and planning.

## 4. Technical and Clinical Success, Patency, and Comparative Outcomes

Early feasibility studies established the procedural safety and reliability of endoAVF creation. The NEAT (Novel Endovascular Access Trial) study reported a technical success rate of 98% for proximal forearm fistulas created with a first-generation 4-Fr system (EverlinQ, no longer marketed), with 87% of accesses reaching ultrasound criteria permissive for cannulation within 90 days [9]. The EASE study, evaluating the 4-Fr WavelinQ device, confirmed a 97% technical success rate and 76% suitability for dialysis use at 6 months, suggesting outcomes at least comparable with historical surgical AVF benchmarks [12]. Real-time intra-procedural imaging permitted immediate confirmation of fistula creation based on spectral Doppler flow analysis; most failures were attributable to inadequate venous outflow rather than device malfunction. Post-marketing registries have extended these findings to larger “real-world” populations. The Ellipsys registry, including 60 patients, reported a 96.7% technical success rate and effective cannulation in 87% of cases at 90 days, with performance maintained at 6 and 12 months [13]. At 12–24 months of follow-up, reported primary patency ranged from 69 to 74%, with secondary patency > 90% and mean blood flow > 700 mL/min; these midterm findings are supported by an extended pivotal-trial follow-up, which suggests sustained usability and durability over a longer horizon when embedded in structured follow-up pathways [14,15]. Data for WavelinQ are broadly consistent with these findings across prospective experience and subsequent clinical adoption [12].

Table 1 summarizes key clinical studies, registries, and comparative series on endoAVF creation. Overall, available clinical evidence suggests that endoAVFs can achieve high technical success and competitive clinical usability compared with surgically created AVFs; however, comparative outcomes (including primary patency and maintenance burden) remain heterogeneous and appear influenced by patient selection and surveillance/maturation protocols. Importantly, head-to-head comparisons between endoAVFs and surgically created AVFs have intrinsic limitations. These accesses are often not truly equivalent interventions, as they may differ in anatomic location, anastomotic configuration, and vessels involved, and endoAVFs are typically created within the deep venous system (with few surgical analogs, aside from selected proximal forearm/Gracz-type configurations). Moreover, access anatomy and maturation trajectories are strongly patient-specific, further constraining the interpretability of nonrandomized comparisons. A meta-analysis by Yan Wee et al. reported high technical success (97.5%) and favorable maturation and patency signals, with pooled 6-month and 12-month patency rates of 92.0% and 85.7%, respectively [20]. A further meta-analysis by Sun et al. (including case series and cohort studies) reported pooled technical success around 98% and pooled maturation around 87%, while comparative cohort data did not show clear differences compared to surgery for procedural success or maturation, underscoring the limitations of nonrandomized evidence [21]. Malik et al. found no significant differences in procedural success (OR 1.44, 95% CI 0.35–5.88), complications (OR 0.28, 95% CI 0.06–1.46), or failure rate; however, they reported pooled data indicated more interventions to maintain patency (OR 1.73, 95% CI 1.22–2.45) and lower primary patency in the endoAVF group (OR 0.34, 95% CI 0.23–0.52) [17]. Beyond pooled analyses, larger post-market and multicenter series have reported sustained usability and favorable cumulative/functional patency signals at extended follow-up, although estimates vary according to surveillance intensity, maturation assistance, and endpoint definitions [22,23,24]. EndoAVFs also exhibit a more predictable maturation timing, with a substantial proportion of accesses reaching two-needle cannulation readiness approximately within 4–8 weeks, depending on flow, diameter, and depth of the cannulation segment [11,13]. In clinical practice, many programs adopt structured Doppler ultrasound surveillance in the first 1–3 months after endoAVF creation, with at least one early follow-up visit within the first 4–6 weeks [14]. Lesions most commonly targeted during this early period include stenosis along the venous outflow tract and competitive branches that siphon flow away from the superficial cannulation segment [14]. Primary or secondary angioplasty and selective embolization with coils or plugs are usually sufficient to restore adequate hemodynamics [14]. Compared with prosthetic grafts, autogenous AVFs generally carry a lower infection risk [1,2]. In major endoAVF trials and registries, access-site infections are uncommon (often ≤1% when reported) [11,22]. Comparative cohorts also report less postoperative discomfort and faster recovery after endoAVF creation than after surgical AVFs, although pain reporting and definitions vary across studies [25,26]. The absence of long skin incisions and prosthetic material likely contributes to the reduced infection risk and greater patient comfort. Direct comparisons between endoAVF and surgically created native AVFs support these observations. Inston et al. reported similar patency rates with WavelinQ-created AVFs and surgical radiocephalic AVFs, but shorter time to cannulation and fewer reinterventions in the endovascular group [25]. In a multicentre trial, Harika et al. found a 96% technical success rate for endoAVFs and 12-month functional patency comparable to surgical radiocephalic AVFs, with a lower angioplasty burden (0.36 vs. 0.68 procedures/patient-year) [27]. Shahverdyan et al. documented similar functional success between Ellipsys-created AVFs and Gracz-type proximal forearm AVFs, with fewer postoperative complications and no access-site infections in an endovascular cohort [28]. In a larger multicentre series, Mordhorst et al. confirmed the non-inferiority of endoAVFs in long-term patency, along with shorter procedure times, minimal anesthesia, and lower postoperative pain scores [26]. Overall, these data suggest that endoAVFs provide patency and clinical usability comparable to surgical AVFs, with, in several domains, signals toward fewer reinterventions, faster recovery, and higher patient comfort, especially when embedded in structured, imaging-guided surveillance programs.

## 5. Complications and Maintenance of Secondary Patency

EndoAVFs exhibit a favorable complication profile, plausibly related to the precision of real-time imaging and the absence of open surgical dissection. Studies report early hematoma and thrombosis rates around 1–3%, while access-site infections and pseudoaneurysms are uncommon (<1%) [11,22]. The main causes of primary dysfunction remain stenosis along the venous outflow tract of the AVF circuit and failure of access maturation (that may be due to inadequate superficialization of flow because of competitive branches or preferential drainage into the deep venous system). These issues are typically manageable by endovascular means, including balloon angioplasty of focal stenoses and selective coil or plug embolization of branches that steal flow from the superficial venous circuit [8]. In published surveillance protocols, several centers schedule early Doppler ultrasound to identify low-flow or poorly maturing accesses (e.g., access flow < 500–600 mL/min or vein diameter < 5–6 mm) and perform pre-emptive interventions before clinical dysfunction or thrombosis occurs [2,8,29,30]; when reported as overall procedural burden across mixed follow-up windows (often including early maturation assistance), secondary intervention rates for endoAVFs generally remain below one procedure per patient-year, with values roughly ranging from 0.4 to 0.9 procedures per patient-year depending on device platform, endpoint definitions, and follow-up intensity [16,17,19,20,25,26,27,28,31]. In surgical cohorts, early thrombosis and non-maturation requiring intervention are strongly associated with reduced cumulative access survival [32,33]. Published endoAVF series, although relatively small and anatomically selected, consistently report low early thrombosis rates (range 1–3%); the need for reintervention to promote maturation (primary angioplasty) or maintain patency (secondary angioplasty) also appears moderate: studies describe secondary intervention rates that remain well below one procedure per patient-year, with values roughly in the range of 0.4–0.9 procedures per patient-year depending on the device, definition, and follow-up window considered [14,15]; for example, in an extended follow-up of endoAVF made by the Ellipsys system reported by Hull et al., the number of procedures performed to maintain function and patency was 0.32 procedures per patient-year during the follow-up windows from year 2 to year 5, suggesting a low late maintenance requirement once the access is established; it is also important to note that no major pAVF-related complications were observed during follow-up [24]. In contrast, classic surgical AVF cohorts often require substantially more reinterventions: Falk et al., for example, reported 209 maintenance procedures in 63 mature autogenous fistulas, corresponding to 1.75 interventions per patient-year to maintain patency [31]. In centers that have implemented structured endoAVF surveillance protocol, including close collaboration between nephrologists, interventionalists, and dialysis staff, high secondary patency rates have been reported up to 2 years after creation [13,15]. These programs typically combine protocol-driven Doppler ultrasound surveillance with proactive management of stenosis and accessory branches. Favourable outcomes observed in these series are in line with the broader vascular access literature, which emphasizes the importance of structured surveillance and timely intervention for optimizing long-term patency [2], and they support the view that endoAVFs can perform robustly when integrated into multidisciplinary care pathways.

## 6. Economic and Organizational Considerations

To the best of our knowledge, no prospective studies have systematically evaluated the economic impact of endovascular AVF creation. Nonetheless, several signals suggest that endoAVFs could confer relevant advantages in resource allocation. Percutaneous access creation in a single session and often on an outpatient basis may reduce inpatient days, decrease dependence on anesthesia resources, and lessen the need for intensive postoperative nursing care [34]. Although device costs for endoAVF systems are higher than those of conventional surgical AVF procedures, these upfront expenses may be partially or fully offset by fewer reinterventions, shorter times to functional dialysis use, and potential reductions in catheter dependence [35]. From an organizational point of view, endoAVF programs require close operational integration between nephrology, interventional radiology, and vascular surgery for access planning and longitudinal management, consistent with modern recommendations that emphasize individualized, imaging-guided strategies [2,7,36]. To date, there are no prospective studies specifically designed to compare the costs of endoAVFs versus surgically created native or prosthetic AVFs in the Italian hospital setting. The learning curve of operators, the need for dedicated equipment and long-term horizons required to capture the full impact of reinterventions, access-related hospitalizations, catheter use, and indirect costs further complicate economic evaluation. Dedicated cost-effectiveness and budget-impact analyses, ideally embedded in pragmatic comparative studies, are needed to fully define the economic profile of endoAVFs [34,35].

## 7. Discussion

### 7.1. What Does the Current Literature Suggest?

In the last decade, endoAVF creation has transitioned from early feasibility experience to routine adoption in selected centers, expanding options for autogenous access in suitable anatomy. Across pivotal trials and post-market registries, technical success is consistently high and clinical usability is achieved in a large proportion of cases [9,11,12,13,15]. At the same time, pooled comparative estimates versus surgery remain heterogeneous across endpoints and study designs, highlighting the limitations of nonrandomized evidence and variability in surveillance and maturation assistance practices [17]. Comparative cohorts generally report broadly similar functional outcomes to surgical AVFs in selected populations, although study designs and endpoints vary [25,26,27,28].

First, percutaneous creation minimizes tissue disruption: it avoids extensive subcutaneous dissection, limits adventitial injury, may better preserve endothelial integrity, and may potentially attenuate inflammatory activation and neointimal hyperplasia—a key driver of early AVF failure [37]. Second, endoAVFs typically result in an anastomotic configuration that may be hemodynamically favorable. Anastomotic geometry influences local shear-stress distribution and downstream remodeling, and specific configurations have been associated with maturation trajectories and reintervention risks [38].

In this context, available evidence does not consistently show clinically meaningful differences in key outcomes between device platforms; however, cross-study comparisons are limited by heterogeneous populations, anatomy, and surveillance protocols [17]. In practice, optimal results depend on rigorous anatomic selection. Current recommendations generally propose minimum diameters ≥ 2 mm for the target artery and vein, an artery–vein distance < 1 mm, a preferred depth < 6 mm, and absence of significant stenosis along the venous outflow axis [8,39]. In daily practice, suitability also depends on the anticipated timing of kidney replacement therapy and patient-specific factors (e.g., obesity, prior access history, central venous stenosis), because access planning must balance timely usability against preservation of future surgical options [1,2,7].

Because endoAVFs use distal segments (such as the proximal radial artery and perforator vein, or paired forearm comitant vessels), they preserve proximal venous capital and keep traditional surgical sites available for future access creation [39]. This vessel-sparing approach is particularly important in younger patients and in those with a long-expected dialysis vintage. In practical terms, an endoAVF can be followed, if needed, by a more proximal AVF (e.g., brachiocephalic), without compromising usual surgical options. The two currently available systems have distinct operational requirements: WavelinQ uses a dual-catheter configuration with magnetic alignment under angiographic guidance, whereas Ellipsys is performed entirely under ultrasound guidance [7,10,22]. Accordingly, device choice is influenced by the availability of an angiography suite, high-resolution ultrasound equipment, and local expertise. In both cases, the learning curve appears relatively short for operators already experienced in interventional dialysis-access procedures [6,7,8].

ECD ultrasound is central in identifying accessory veins that divert flow, juxta-anastomotic stenoses, or suboptimal depth of the intended cannulation segment [2,39]. When indicated, primary or secondary angioplasty and selective embolization can help optimize hemodynamics [19,39]; in some comparative cohorts, the overall intervention burden and time to usability appear favorable compared to surgery [25,27]. Ultrasound-guided cannulation, when implemented, may facilitate safe cannulation in challenging anatomy [2]. Observational comparisons suggest broadly similar 12-month functional patency between endoAVF and surgical AVF, while time to maturation/usability may favor endoAVFs in selected cohorts [25,26].

Systematic reviews and meta-analyses generally confirm high technical success and clinically relevant maturation rates. However, comparative pooled estimates versus surgery remain discordant across endpoints. Some analyses suggest shorter procedural times but more maintenance interventions and lower primary patency for endoAVF, highlighting the impact of study design, endpoint definitions, and local surveillance protocols [20,21]. Sun et al. also report substantial heterogeneity in included studies (design, patient selection, follow-up, and endpoints), which limits direct comparability both across endoAVF series and versus surgical case series [21].

From a methodological point of view, most comparative evidence comes from single-center observational cohorts in selected populations, so residual confounding and selection bias remain likely. Future studies should improve transparency and comparability through standardized reporting and explicit appraisal of study quality [40]. Heterogeneity in device platforms, ECD ultrasound surveillance protocols, cannulation practices, and population characteristics should be reported transparently and quantified using standard heterogeneity metrics [41]. Equally important is the consistent adoption of consensus endpoint definitions for vascular access research (primary, primary-assisted, and secondary patency; technical and clinical success) [18,21]. Standardized outcomes would make cross-study comparisons more reliable and strengthen future pooled analyses.

Overall, mechanistic rationale and clinical evidence support endoAVFs as a percutaneous, vessel-preserving strategy that can deliver reliable technical success, low complication rates, and competitive patency in appropriately selected patients. Maintenance requirements appear protocol- and setting-dependent, with several cohorts suggesting favorable profiles, while pooled comparative analyses report heterogeneous results, including the possibility of greater maintenance burden and lower primary patency [23,24]. Within multidisciplinary pathways, with careful selection and structured surveillance, endoAVF creation may be considered a front-line option in anatomically suitable candidates with suboptimal distal venous capital, complementing rather than replacing surgery and potentially helping preserve proximal venous pathways throughout the patient’s lifetime [39].

### 7.2. What Are the Practical Implications?

Implementation of endoAVF programs relies on structured patient selection, pre-procedural vascular mapping, early ECD ultrasound follow-up, and proactive maturation assistance when indicated. Contemporary society practice guidelines provide a pragmatic approach regarding set-up, follow-up, and troubleshooting. In contemporary access planning, endoAVFs should be framed within a patient-centered “ESKD life-plan” and a distal-first strategy [2,39].

EndoAVFs should not be viewed as a direct replacement for surgery, but rather as an additional interventional option that enables use of a mid-arm vascular territory previously considered marginal. Through a minimally invasive, vessel-sparing approach, endoAVFs may increase the likelihood of autologous access creation while preserving more proximal venous and arterial vessels for subsequent surgical options, including prosthetic bridges, when indicated [39]. Clinically, they are particularly relevant when distal superficial vessels are unsuitable for sAVF creation, but deep venous/perforator anatomy is favorable [39].

In patients with limited distal vascular capital, endoAVF creation should be considered when minimum anatomic criteria are satisfied, including perforator vein and target artery diameters ≥ 2 mm and close artery–vein proximity (<1 mm for WavelinQ; ≤1.5 mm for Ellipsys). Absolute exclusion includes absence of a suitable perforating vein, while central venous stenosis or lack of a cannulable superficial outflow segment may limit feasibility and should be addressed during planning [8,39].

In late 2025, the Society of Interventional Radiology (SIR) issued an expert consensus-based practice guidance to standardize the key requirements for developing and running an endoAVF program, covering patient preparation, procedural performance, adverse event management, and structured follow-up [39]. The document stresses rigorous patient selection supported by detailed vascular assessment: history should document prior central venous devices and previous vascular accesses, and clinical evaluation should include palmar arch assessment. Pre-procedural mapping is considered mandatory and should characterize inflow arteries, perforator vein(s), and superficial outflow veins; it also reiterates practical anatomic targets (perforator vein diameter ≥ 2 mm and an intended cannulation segment no deeper than 6 mm). Even in this recent guidance, endoAVFs are framed within a “distal-first” strategy, with surgical options at the distal third of the upper limb (including the wrist) remaining preferable when feasible. Regarding maturation and complications, SIR notes that technical success is typically high, yet adjunctive maturation or corrective procedures are common; published series report overall maturation rates of about 80%, which should be explicitly addressed during informed consent. For accesses that fail to mature within the expected timeframe, the consensus recommends an algorithmic pathway including angioplasty of inflow or outflow stenoses and, when deep venous diversion is present, selective embolization of the deep venous system or venous ligation to redirect flow toward the superficial cannulation axis [39]. Finally, SIR identifies cannulation as a critical operational step. Because endoAVFs may present differently from surgical fistulas and challenge dialysis teams unfamiliar with them, targeted staff training (including ultrasound-guided cannulation where available) and a clear troubleshooting pathway are recommended, with the interventionalist suld serve as the primary point of contact for cannulation difficulties or suspected complications [39].

When surgical distal radiocephalic AVF is feasible and likely to mature, it remains the simplest and most appropriate distal-first option. Conversely, when the distal superficial venous system is inadequate but the anatomy of the deep (and proximal superficial) and perforating venous system is favorable, endoAVF offers a pragmatic, vessel-sparing alternative integrated within multidisciplinary pathways supported by ECD surveillance and maturation assistance. In this context, access planning evolves from a single procedural decision into a longitudinal strategy spanning the patient’s dialysis lifetime.

### 7.3. Limitations and Clinical Relevance

This review has limitations inherent to its narrative design: we did not perform a systematic study selection process, formal risk-of-bias assessment, or quantitative pooling. Available evidence is dominated by observational cohorts in selected anatomy and experienced centers, with heterogeneity in surveillance intensity, maturation assistance protocols, cannulation practices, and endpoint definitions; therefore, causal inferences and cross-study comparisons should be interpreted cautiously.

Despite these limitations, the present synthesis is intended to support clinical decision-making by summarizing the most consistent signals across trials, registries, and comparative cohorts, and by outlining practical steps for selection, follow-up, and troubleshooting that may facilitate safer and more standardized implementation of endoAVF programs.

## 8. Conclusions

Current evidence supports endoAVF creation as a minimally invasive, vein-sparing strategy capable of achieving high technical success and low rates of major procedural complications. In many cohorts, clinical usability can be achieved within a few weeks when structured ultrasound surveillance and proactive, protocol-driven endovascular maturation assistance interventions are available; however, adjunctive procedures are common and should be anticipated in vascular access planning and explicitly addressed during informed consent; longer-horizon data further suggest that, once established within structured pathways and follow-up, late maintenance requirements may be modest [24]. From an access-planning perspective, endoAVFs should be framed within a patient-centered distal-first life-plan: when a surgical wrist AVF is feasible and likely to mature it remains a strong first option, whereas endoAVF is a pragmatic first-line autogenous alternative when distal superficial venous capital is limited yet deep/perforator anatomy is favorable, preserving proximal options over the patient’s access lifetime [39]. Primary-assisted and secondary functional patency outcomes are generally competitive with surgically created AVFs in selected patients, while pooled comparative analyses highlight heterogeneity across endpoints and suggest that, in some settings, primary patency and maintenance burden may be less favorable for endoAVFs. From an access-planning perspective, use of distal vascular segments and preservation of proximal venous pathways may confer a strategic lifetime advantage. Organizationally, integrating endoAVFs into shared pathways involving nephrology, interventional radiology, and vascular surgery is consistent with imaging-guided models for access selection and longitudinal management and is supported by recent consensus practice guidance and evidence, which emphasize standardized mapping, expectation setting, troubleshooting algorithms, and dialysis-unit cannulation training. Economic implications remain uncertain, likely heterogeneous, and strongly context-dependent; available models suggest a potentially competitive long-term cost profile versus surgery, but robust conclusions require prospective, healthcare-system-specific evaluations [34,35]. Some important knowledge gaps persist. Future research should prioritize pragmatic comparative studies and long-term registries using standardized endpoint definitions [16,18] to assess catheter dependence, access-related hospitalizations, patient-reported outcomes, and total costs, ideally within specific healthcare systems (including the Italian setting). In the interim, endoAVFs should be considered a front-line vascular access option for patients with favorable anatomy, including those with suboptimal distal venous capital, treated in experienced centers, complementing rather than replacing surgical strategies within a patient-centered, distal-first access plan. In practical terms, the framework provided here may help multidisciplinary teams set realistic maturation expectations, anticipate the need for maturation assistance, and integrate endoAVFs within a distal-first access strategy while preserving future surgical options.

## Figures and Tables

**Table 1:** Key clinical studies and reviews on endovascular arteriovenous fistula (endoAVF) creation.

First Author	Year	Ref.	Design & Population	Device/Comparison	Main Outcomes & Conclusions
Lok	2017	[9]	Prospective, multicenter trial, *n* = 60	EverlinQ 4-Fr RF system (First-generation)	Technical success 98%; 87% physiologically suitable for dialysis within 3 months; 12-month primary patency 69% and cumulative patency 84%; serious procedure-related AE 8% (2% device-related AE)
Berland	2019	[12]	Prospective, multi-center, *n* = 32	WavelinQ 4 Fr	Technical success 100%; primary patency 83.3% and cumulative patency 86.9% (6 months); 2-needle cannulation 78% within 3 months, procedure-related adverse event 3%.
Hull	2018	[11]	Prospective, multicenter, *n* = 107	Ellipsys 6-Fr	Technical success 95%; primary ECD endpoints achieved 86.0% (92/107); no major device-related AEs; cumulative patency 91.6%/89.3%/86.7% at 90/180/360 days; 2-needle dialysis 88% (71/81) at mean 114.3 ± 66.2 days; functional patency 98.4%/98.4%/92.3% at 90/180/360 days
Hull	2020	[13]	Prospective, *n* = 60	Ellipsys 6 Fr	Technical success 96.7%; 87% 2-needle cannulated by 90 days; 67% required primary intervention for maturation at 4 w, abandonment 5%, procedure-related complication 2.3%.
Mallios	2020	[14]	Retrospective, single-center, *n* = 234	Ellipsys 6 Fr	Technical success 99%, 1-year primary patency 54%, assisted patency 85%, secondary patency 96%,
Beathard	2020	[15]	Prospective, multicenter, *n* = 105	EndoAVF (mixed devices)	Physiologically mature AVF 98%, clinically functional AVF 95%; reported cumulative patency at 6/12/18/24 mo = 97.1%/93.9%/93.9%/92.7%.
Berland	2022	[22]	Multicentre, post hoc analysis, *n* = 120	WavelinQ 4 Fr	Procedural success 96.7%; 6-mo patency: primary 71.9%, assisted-primary 80.7%, secondary 87.8%; device-related adverse events 2.5% (3/120), procedure-related adverse events 5.8% (7/120); no arterial/venous access complications
Inston	2020	[25]	Prospective, single-center, *n* = 30 (vs. sAVF *n* = 40)	WavelinQ 4 Fr vs. surgical radiocephalic AVF	Technical success endoAVF 96.7% vs. sAVF 92.6%, primary patency endoAVF 65.5% vs. sAVF 53.4% at 6 months follow-up; mean primary patency was significantly lower for sAVF vs. endoAVF
Harika	2021	[27]	Comparative, retrospective, single-center, (endoAVF *n* = 107 vs. sAVF *n* = 107)	Ellipsys 6 Fr	EndoAVF had higher 6-week maturation (65% vs. 50%), while sAVF had higher 12-month primary patency (86% vs. 61%); primary patency was similar at 24 months (52% vs. 55%). Secondary patency was similar at 12/24 months (90–91%/88–91%)
Shahverdyan	2021	[28]	Comparative, endoAVF *n* = 89 vs. sAVF *n* = 69)	Ellipsys 6 Fr vs. proximal forearm Gracz sAVF	Technical success was 100% in both groups; at 12 months, primary patency failure tended to be lower with sAVF (47% vs. 64%; *p* = 0.1) and secondary patency failure was similar (20% vs. 12%; *p* = 0.3). In PRA sAVFs, primary patency was similar (65% vs. 64%), but secondary patency failure was higher than endoAVF (34% vs. 12%; *p* = 0.04)
Mordhorst	2022	[26]	Retrospective, comparative, multicentre, endoAVF *n* = 61 vs. sAVF *n* = 308 (RC + BC)	sAVF vs. endoAVF	Primary patency at 12/24 mo: endoAVF 42% ± 5/32% ± 7, RC sAVF 43% ± 4/24% ± 4, BC sAVF 42% ± 4/29% ± 4 (*p* = 0.906). Secondary patency at 12/24 mo: endoAVF 68% ± 6/60% ± 7, RC sAVF 75% ± 3/67% ± 4, BC sAVF 91% ± 3/81% ± 4; Steal syndrome and reinterventions/year were similar across groups
Yan Wee	2020	[20]	Systematic review and meta-analysis, *n* = 300	EndoAVF (mixed devices)	Technical success 97.50%, 90-day maturation 89.3%; patency 91.99% at 6 months and 85.71% at 12 months; procedure-related complications 5.46%. Meta-regression: age, diabetes, race, hypertension, dialysis status, and BMI did not explain heterogeneity
Malik	2022	[17]	Systematic review and meta-analysis, *n* = 527	EndoAVF (mixed device) vs. sAVF	No significant difference in procedural success (OR 1.44, 95% CI 0.35–5.88) or complications (OR 0.28, 0.06–1.46) or failure rate (OR 1.03, 0.21–5.13). Significant differences reported for further interventions (OR 1.73, 1.22–2.45) and primary patency (OR 0.34, 0.23–0.52)
Hull	2022	[24]	Retrospective, *n* = 85	Ellipsys 6 Fr	Maturation 99%; 99% of dialysis patients used pAVF; maintenance required in 31.8% (dysfunction 21.2%, thrombosis 5.9%, cannulation injury 12.9%, arm swelling 4.7%); procedures/patient-year to maintain function/patency 0.32 in years 2–5. Cumulative patency years 1–5: 89.5%, 88.4%, 88.4%, 85.6%, 82.0%; functional patency 91.8% at study end; no major pAVF-related complications
Sun	2022	[21]	Systematic review/meta-analysis, *n* = 1929	EndoAVF (mixed device) vs. sAVF	Technical success (endoAVF): 98.0% (95% CI). No difference vs. sAVF for procedural success: OR 0.69 (95% CI). Maturation (endoAVF): 87.0% (95% CI 0.79–0.93; I^2^ = 83.96%); no difference vs. sAVF (3 cohorts): OR 0.73 (95% CI 0.20–2.63; *p* = 0.63; I^2^ = 88%).
Klein	2024	[23]	Retrospective, multi-center, *n* = 112	EndoAVF, WavelinQ	Technical success 97.3%; maturation 87% (98/112), non-maturation 11/112. Cumulative patency at 12/24 mo: 94.3%/91.7%; functional patency (2-needle) at 12/24 mo: 95.7%/92.7%. Median maturation time 95 days (IQR 51–231); predictors of maturation: male sex and brachial vein coiling. Reinterventions/patient-year 0.73, complication: 3 patients

## Data Availability

No new data were created or analyzed in this study.

## Abbreviations

List of abbreviations: AE, adverse event; AVF, arteriovenous fistula; BC, brachiocephalic (fistula); BMI, body mass index; CI, confidence interval; CVC, central venous catheter; DRG, diagnosis-related group; EASE, Endovascular Access Study; ECD, echo-color Doppler (duplex ultrasound); endoAVF, endovascular arteriovenous fistula; ESKD, end-stage kidney disease; IQR, interquartile range; KDOQI, Kidney Disease Outcomes Quality Initiative; mo, months; NEAT, Novel Endovascular Access Trial; OR, odds ratio; pAVF, percutaneous arteriovenous fistula; PRA, proximal radial artery; PRISMA, Preferred Reporting Items for Systematic Reviews and Meta-Analyses; RC, radiocephalic (fistula); RF, radiofrequency; sAVF, surgically created arteriovenous fistula; SIR, Society of Interventional Radiology; VAS, visual analog scale.
